# An Analytical Model for Interference Alignment in Broadcast Assisted VANETs

**DOI:** 10.3390/s19224988

**Published:** 2019-11-15

**Authors:** Chong Zhao, Jianghong Han, Xu Ding, Lei Shi, Fan Yang

**Affiliations:** 1 School of Computer and Information, Hefei University of Technology, Heifei 23009, China; zhaochong@mail.hfut.edu.cn (C.Z.); hanjh@hfut.edu.cn (J.H.); shilei@hfut.edu.cn (L.S.); yangfan@mail.hfut.edu.cn (F.Y.); 2 Institute of Industry and Equipment Technology, Hefei University of Technology, Hefei 230009, China

**Keywords:** interference alignment, modeling and optimization, VANETs

## Abstract

Application of safety-related information interaction among vehicles has always been a research frontier in Vehicular Ad-hoc NETworks (VANETs). These messages require high real-time performance. There is a lot of research dependant on creating optimization model for communication task scheduling or routing protocols to reduce communication delay. In this paper, we analyze characteristics of safety-related information and introduce Interference Alignment (IA) technology in VANETs. To further improve routing efficiency, a data-driven assisted transmission routing and broadcast model framework for Vehicle to Vehicle(V2V) and Vehicle to Infrastructure (V2I) communication are constructed which are the basis for IA. Depending on the proposed model, we propose an optimization problem of minimizing total number of time slots required for safety information sharing in VANETs. Then a clustering algorithm is designed to narrow feasible solution space. Simulation results show that the approach can effectively reduce the number of time slots required and improve link use by 20% percent compared with no IA applied.

## 1. Introduction

The US Federal Communications Commission (FCC) allocated 75 MHz of wireless spectrum (5.850 GHz to 5.925 GHz) bandwidth to dedicated short range communications (DSRC) for intelligent transportation systems (ITS) service. According to FCC regulations, this band is further divided into seven isolated sub-channels, including one control channel and six service channels, where control channel is reserved for transmitting beacons or fundamental safety-related messages. Each vehicle periodically broadcasts traffic state information including its speed, acceleration and GPS on control channel. These types of information help vehicle interacts with surrounding vehicles timely. However, with the number of vehicles increasing, demand for communication between vehicles rises, causing congestion in communication band and decreasing communication efficiency.

Communication protocols of VANETs are mainly based on IEEE802.11 series protocols, but a large number of performance verification experiments show that competitive protocols cannot meet Quality of communication Service (QoS) requirement of VANETs [1,2]. Since safety related information should be delivered as quickly as possible, the effective method is that vehicles broadcast their safety-related information directly to surrounding vehicles, which will inevitably lead to information redundancy and flooding [3].

In the area of wireless communication networks, some research was conducted to improve channel resource use though managing interference between wireless terminals, such as Interference Alignment (IA). Simulation results in [4,5] show that in interference channel of the K-node communication network equipped with M antennas, if IA is adopted, the total degree of whole network freedom approaches KM/2. However, Channel State Information (CSI) of senders and receivers are required for IA application. The relative positions of vehicles are constantly changing, making it difficult to obtain CSI information.

Since the location of base station is fixed, in some studies, prediction model of CSI between vehicle with base station are constructed to estimate CSI during a certain period of time [6,7]. Once CSI information between vehicles and base station can be predicted in advance, IA can be applied to communication of V2I.

With the advent of Cognitive Radio (CR) and dynamic spectrum access technology, Spectrum Database (SD) including CSI information provided by ITS could be obtained by a user who is about to pass a road segment [8]. Depending on SD and the user’s location, CSI between the user and base station could be queried, which could meet time requirement for safety related information in a long enough period [9].

All above, in this paper, we analyze the communication flow of V2V and V2I in VANETs and construct broadcast model for IA technology. Different communication fashion is adopt for different type senders. Combined with characteristics of safety related information, an assisted transmission model is constructed to further reduce redundancy transmission and improve link use efficiency. Based on these model, we propose an optimization problem of minimizing total number of time slots required for safety information sharing in VANETs. In addition, a clustering algorithm is designed to narrow feasible solution space.

### 1.1. Literature Review

Safety related information exchange is the key to the next generation of VANETs. In VANETs, vehicle is required to exchange information quickly and reliably with others within safety radius. In order to fulfill this target, some studies have designed VANETs distributed cross-layer protocols to adjust the priority of safety related information and intervene the direction of data propagation to reduce communication delay [10]. In [11], Velmurugan et al., proposed a new algorithm works on the selective distance allocation methodology for data transmission.

As an emerging multiplexing-based interference cancellation method, IA is based on use of channel transmission features to align multiple interference streams to a specific direction at the receiving node to reduce interference. In [12], sAhn et al., proposed a new cellular network interference management method, which applied IA to cellular network. The simulation verified that IA can effectively improve communication rate. There is some research that studied a blind IA in cell network without full CSI information [13,14,15]. However, without full CSI information, the blind IA caused a large delay in direct communication and relatively large loss of effectiveness in routing.

In [16], Liang et al., proposed an intra-group IA scheme for V2V. In their research, the network is divided into two groups. One group used doppler frequency domain IA to ensure vehicles in this group are not interfered by other same group vehicles, while other groups only experience partial interference. However, because of the mobility, the vehicle cluster couldn’t keep for a long time.

In [17], Cheng et al., combined VANETs with CR called CR-VANETs protocol to solve spectrum scarcity problem. This method significantly increased spectrum use by equipping vehicles with a CR communication device to detect idle channel in DSRC. With the ability to capture and use available CR spectrum holes in space and time, vehicles were able to make full use of spectrum resources, thereby improving communication efficiency of VANETs [18,19].

In contrast to the preemptive channel exclusive method such as IEEE802.11p, i.e., in IA application, channel resources are allocated dynamically and the channel access method is time division multiple access (TDMA) [20,21]. There are a lot of researchers that apply TDMA in VANETs and prove TDMA could overcome problems of hidden and exposed terminals. Meanwhile, TDMA makes it possible to facilitate IA in VANETs. The study in [22] verifies the feasibility of TDMA in VANETs and proves that TDMA has shorter latency and higher communication efficiency than IEEE802.11p.

Most of the broadcast models are developed from a periodic single-hop communication. In [23], Kang et al., proposed a new model to calculate a single-hop survival broadcast packet probability with a forwarding mechanism. This model accounted all possible cases of contention window assignments to all the nodes simultaneously receiving a broadcast message. Without considering characteristic of safety related information, the performance would be limited by bandwidth. For piggybacking traffic information over periodic safety related messages, Abbasi et al., proposed a highly efficient and reliable multi-hop broadcasting protocol, Intelligent Forwarding Protocol (IFP) [24]. Tahmasbi-Sarvestani proposed a network-aware double-layer distance-dependent protocol for fast broadcasting of aggregated traffic information over multiple hops [25]. However, the hidden and exposed terminal problems still remained in these researches.

### 1.2. Goals of This Paper and Main Contributions

The main contributions of this article are as follows:

- Depending on analyzing communication flow between base station and vehicles in VANETs, we construct V2V and V2I broadcast model. In this model, IA technology is adopted in upload transmission for V2I in VANETs.
- Combining characteristics of safety-related information, we build a data-driven assisted transmission model to improve link reuse rate.
- We propose an optimization problem of minimizing total number of time slots required for safety information sharing in VANETs. In order to solve this problem, we reform constraints in optimization problem by transforming quadratic items into multiple linear constraints, which simplify the optimization problem.

Based on this model, solution results of the optimization problem show that data-driven assisted transmission-based IA Application can effectively improve link reuse rate and reduce communication delay in VANETs.

### 1.3. Organization of this Paper

The remainder of this article is organized as follows: Section 1 introduces related works. Section 2 analyzes the research and communication scenario of VANETs, explains symbols in this paper and communication procedure. In Section 3, V2V & V2I broadcast model and IA model are constructed in detail. Combined with the characteristics of safety-related information, VANETs data-driven assisted transmission model is constructed and the optimization problem is proposed. Section 4 designs a clustering algorithm to pre-cut feasible solution space for optimization problem in Section 3. In Section 5, results of experiments are presented to evaluate performance of our approach. Section 6 concludes this paper.

## 2. System Model

In VANETs, according to the type of message sender and receiver, the communication fashions can be divided into two categories: (1). Vehicle to Vehicle (V2V), which means the sender and receiver are all vehicles; 2. Vehicle to infrastructure (V2I). In (V2I), the types of terminals include Base Station (BS), wireless access point (AP) and other infrastructure. Without loss of generality, in this paper, we use *V* to indicate vehicle and *I* as BS, AP, etc.

### 2.1. Research Scenario in VANETs and the Symbol System

**Figure 1:**
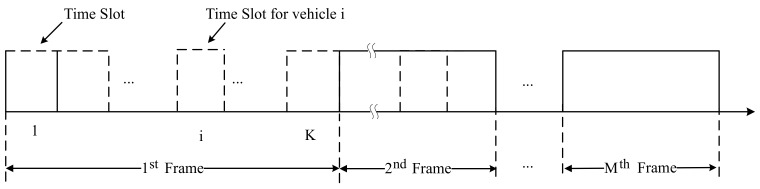
Contents of Scheduling Time.

As shown in Figure 1, the entire VANETs scheduling time can be divided into several time frames. Every frame is taken as one scheduling period containing several equal interval Time Slots (TS) based on TDMA. Each vehicle transmits data during the assigned TS. However, before the scheduling period, the base station sends the status of the entire network and TS scheduling scheme to each vehicle to avoid communication collision.

- In this scenario, both V2V and V2I communication fashion are coexisting simultaneously.
- In this scenario, the vehicle speed is relatively low and SD could be updated timely.
- Due to the buildings and other architectures, CSI amongst vehicles with base station varies a lot, which should be solved first of all.

**Figure 2:**
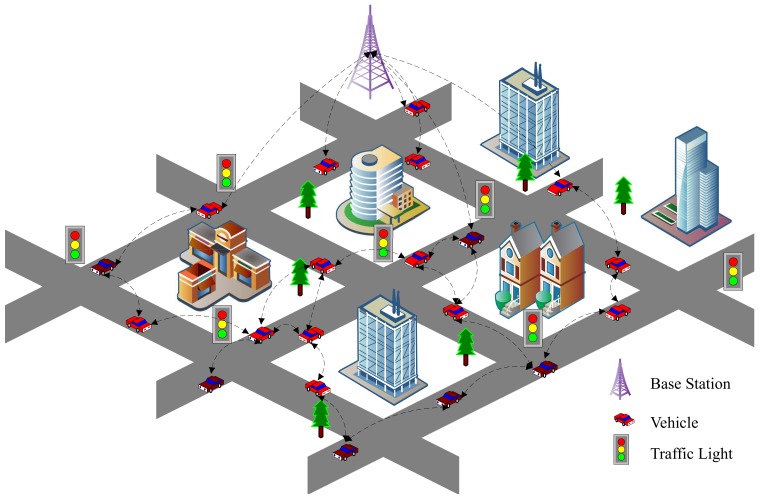
Typical VANETs Scenario.

In this road section, there is one base station and several vehicles, denoted by *I* and *V* respectively in this paper as shown in Figure 2.

Symbol I→V indicates base station broadcasts message to vehicle and V→I indicates vehicles upload information to Base Station. Vehicle communicates with each other denoted as V⇌V. Other symbols in this paper are listed in Table 1. To simply formulas, we use i=1:N to represent 1≤i≤N, in which *i* is integer.

### 2.2. Case Study in VANETs Communication

CSI is the key data for IA technology application. In VANETs, CSI could be obtained from SD in ITS and should be updated in time. When a vehicle enters a new road, firstly, it registers itself in VANETs. If the vehicle is within communication range of base station, the registration request could be posted to base station directly. Otherwise the vehicle should turn to other vehicles for relaying the request. After registration, base station allocates idle time slot and sends SD containing CSI of this road to the new vehicle.

**Figure 3:**
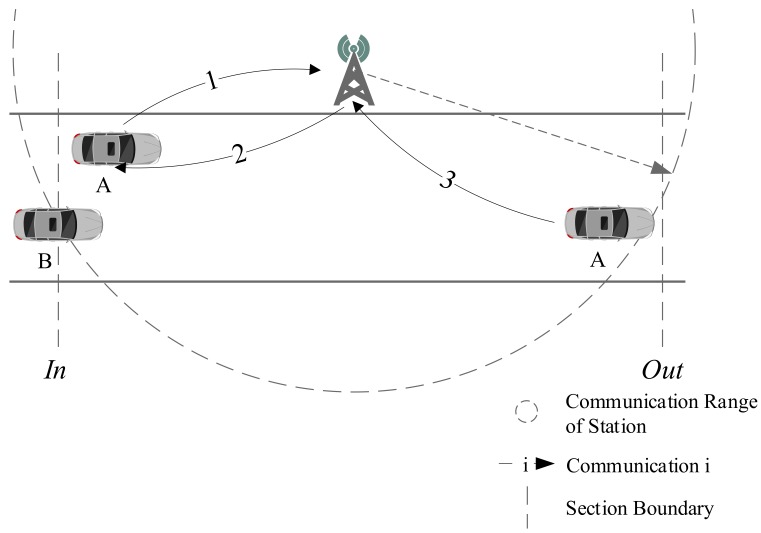
Workflow of New Vehicle Registering at Base Station and Uploading CSI to SD.

The detailed procedure is shown in Figure 3, which contains 4 stages. In the first stage, when vehicle *A* enters the range of base station from the boundary ***In*** point, it posts an access request to base station in communication 1. During this stage, the base station collects the new access request and allots idle time slot to vehicle *A*. During the second stage, the base station sends SD and time slot information to vehicle *A* in communication 2. During the third stage, vehicle *A* runs on this road and collects CSI with base station. At the last stage, when vehicle *A* reaches the boundary ***Out*** point, the latest CSI information collected by vehicle *A* is sent to base station in communication 3 for SD update. When a new vehicle *B* enters this road, it will repeat this process as vehicle *A*. In this way, the base station could keep the latest SD of this road.

**Figure 4:**
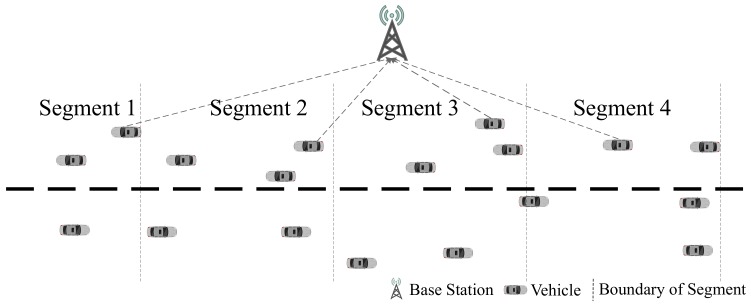
CSI Based Road Segments.

During stage 3, vehicles broadcast their safety related information to surrounding vehicles. These information should be pre-encoded based on CSI before being broadcast if IA is adopt. After the information is encoded with pre-coded vector, the valid signals are mapped to different directions with the interference signals [4]. If the road is reasonably divided, as shown in Figure 4, the CSI in the same segment can be considered as the same value [10].

In this paper, vehicles adopts half-duplex working mode and safety-related information contains location, speed and acceleration et al., In VANETs, the base station is equipped with multiple antennas for Multiple-Input Multiple-Output (MIMO). A communication task that sending a safety related information from one vehicle to another one, is called a communication session. The fashions of communication sessions sent from different senders to receivers can be categorized as follows:

I→V: For base station, it sends information to multiple vehicles simultaneously by broadcast.

V→I: Since the base station is fixed, vehicles can obtain CSI from SD, thereby making it possible to implement IA MIMO communication with base station.

V⇌V: Due to vehicle mobility, CSI between vehicles is not stable and difficult to be maintained. For this reason, broadcast is used as communication method for V2V without IA.

**Figure 5:**
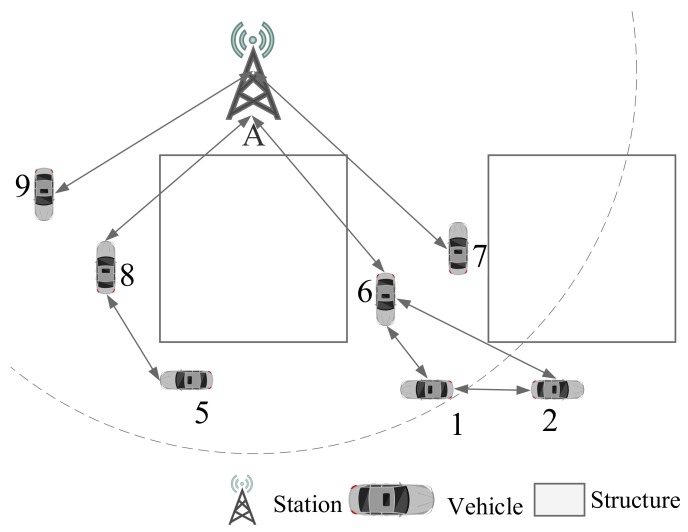
Illustration of Base Station Broadcast Coverage and Assisted Transmission.

In addition, due to limited coverage of base station, the vehicles among communication range could communicate with base station directly. As shown in Figure 5, coverage of the communication for all vehicles cannot be achieved. Such as the vehicle 2 is out of base station’s service. For these vehicles, they should be assisted to upload information to base station.

## 3. Model of Broadcast Assisted Transmission Based IA Application in VANETs

In this section, we construct IA application model in VANETs. At first, V2V and V2I broadcast model is constructed as basis for IA. Then IA constraints are formulated. In order to support multi-hop, a data-driven assisted transmission model is constructed according to characteristics of safety related information.

### 3.1. V2V and V2I Broadcast Model

In our paper, all terminals adopt a half-duplex mode of operation to switch between receiving and transmitting state. $\delta {\;}_{T}^{i}\left(t\right)$ is the sending state of vehicle *i* at time slot *t* and $\delta {\;}_{R}^{i}\left(t\right)$ is receiving state. Then we get

(1) $$\left\{\begin{array}{c} 0\leq \delta {\;}_{T}^{i}\left(t\right)\leq 1\\ 0\leq \delta {\;}_{R}^{i}\left(t\right)\leq 1 \end{array}\right.,\;\left(1\leq i\leq N,1\leq t\leq K\right)$$

(2) $$\delta {\;}_{T}^{i}\left(t\right)+\delta {\;}_{R}^{i}\left(t\right)\leq 1,\;\left(1\leq i\leq N,1\leq t\leq K\right)$$

Since all vehicles should broadcast their safety related information in one scheduling frame, which means they must make a broadcast task during. Thus, we have

(3) $$\left\{\begin{array}{c} \sum_{t=1:K}\delta {\;}_{T}^{i}\left(t\right)\geq 1\\ \sum_{t=1:K,j=1:N}\lambda {\;}_{i,j}\left(t\right)\geq 1,\left(L\left(i,j\right)=1\right) \end{array},\;\left(i\in Src\right)\right.$$

For V2V communication, IA cannot be applied, so the number of valid session and interference received by a vehicle cannot exceed the number of its antennas [8]. When a vehicle broadcasts to surrounding vehicles, number of receivers is equal to the number of vehicles in receiving state. Thus, the following equation should be met:

(4) $$\left\{\begin{array}{c} \sum_{j=1:N}\lambda {\;}_{i,j}\left(t\right)\leq \sum_{j=1:N}L\left(i,j\right)\cdot \delta {\;}_{R}^{j}\left(t\right),\left(\delta {\;}_{T}^{i}\left(t\right)=1\right)\\ \sum_{j=1:N}\lambda {\;}_{i,j}\left(t\right)=0,\left(\delta_{T}^{i}\left(t\right)=0\right) \end{array}\right.$$

Constraints in (4) can be further combined as (5):

(5) $$\begin{array}{c} \sum_{j=1:N}\lambda {\;}_{i,j}^{t}\leq \delta {\;}_{T}^{i}\left(t\right)\cdot \sum_{j=1:N}\left[L\left(i,j\right)\cdot \delta {\;}_{R}^{j}\left(t\right)\right]\\ \Rightarrow \left\{\begin{array}{c} \sum_{j=1:N}\lambda {\;}_{i,j}^{t}\leq \sum_{j=1:N}\left[L\left(i,j\right)\cdot \delta {\;}_{R}^{j}\left(t\right)\right]\\ \sum_{j=1:N}\lambda {\;}_{i,j}^{t}\leq N_{a}\left(i\right)\cdot \delta {\;}_{T}^{i}\left(t\right) \end{array}\right. \end{array}$$

Since vehicle *i* may receive interference from its surrounding vehicles at the same time, the intended information might not be decoded. Therefore, at the same time, the amount of vehicle data received by vehicle *i* cannot exceed its number of antennas, then we get

(6) $$\left\{\begin{array}{c} \sum_{1\leq j\leq N}^{i\neq j}\lambda {\;}_{j,i}\left(t\right)\leq N_{a}\left(i\right),\left(\delta {\;}_{R}^{i}\left(t\right)=1\right)\\ \sum_{1\leq j\leq N}^{i\neq j}\lambda {\;}_{j,i}\left(t\right)=0,\left(\delta {\;}_{R}^{i}\left(t\right)=0\right) \end{array},\left(i\neq St\right)\right.$$

which can be combined as

(7) $$\sum_{1\leq j\leq N}^{i\neq j}\lambda {\;}_{j,i}\left(t\right)\leq \delta {\;}_{R}^{i}\left(t\right)\cdot N_{a}\left(i\right),\;\left(i\neq St\right)$$

When a vehicle is in sending or silent state, and it cannot receive sessions from neighboring vehicles, then the following constraint should be met:

(8) $$\begin{array}{c} \left\{\begin{array}{c} \lambda {\;}_{i,j}\left(t\right)\leq N_{a}\left(i\right)\cdot \delta {\;}_{T}^{i}\left(t\right)\\ \delta {\;}_{T}^{i}\left(t\right)\leq \sum_{t=1:K,j=1:N}\lambda_{i,j}\left(t\right) \end{array},\left(L\left(i,j\right)=1\right)\right. \end{array}$$

If a terminal switches to receiving mode and there is no vehicle sends message to it, it will only receive interference or nothing. Thus, we get

(9) $$\left\{\begin{array}{c} \sum_{j=1:N}\lambda {\;}_{i,j}\left(t\right)\leq \sum_{j=1:N}\delta {\;}_{R}^{j}\left(t\right)\\ \delta {\;}_{R}^{j}\left(t\right)\leq \sum_{j=1:N}\lambda {\;}_{i,j}\left(t\right) \end{array}\right.,\left(i\neq St,L\left(i,j\right)=1\right)$$

For a vehicle in receiving state, the number of interference a vehicle can receive is no exceed the number of its antennas. Then we get

(10) $$il{}_{i,j}\left(t\right)\leq \delta {\;}_{T}^{i}\left(t\right)\cdot N_{a}\left(i\right),\;\left(j\neq St\right)$$

Some constraints can be set default value in advance based on practical situation. When the distance between vehicles exceeds communication range, $\lambda_{i,j}\left(t\right)$ equals 0. Similarly, when vehicles out of interference range with each others, there is no interference. Then, we have the following equations

(11) $$\left\{\begin{array}{c} \lambda {\;}_{i,j}\left(t\right)\leq N_{a}\left(i\right)\cdot L\left(i,j\right)\\ il_{i,j}\left(t\right)\leq N_{a}\left(i\right)\cdot IL\left(i,j\right)\\ al_{i,j}\left(t\right)\leq N_{a}\left(i\right)\cdot IL\left(i,j\right) \end{array}\right.$$

### 3.2. Model of Interference Alignment between Vehicles and Base Station

According to IA technology, when vehicle communicates with base station, the interference caused by other vehicles could be aligned together in the same direction to save network freedom. Since interference from the same CSI cluster cannot be aligned in the same direction [9], then for a cluster *m*, we have following equation:

(12) $$\sum_{i\in C(m)}al_{i,j}\left(t\right)=0,\;\left(j=St\right)$$

Since interference from vehicles in the same cluster cannot be aligned to each other, the number of valid signals and interference caused by vehicles in all areas should be less than the number of antennas of base station. Then, we have

(13) $$\sum_{i\in C(m)}\lambda {\;}_{i,j}\left(t\right)+\sum_{i\in C(m)}il{}_{i,j}\left(t\right)\leq N_{a}\left(j\right),\;\left(j=St\right)$$

The number of interference can be aligned in same cluster cannot outnumber the total number of interference invoked in same area, we get the first constraint in (14). And the actual number of degrees of freedom consumed at base station cannot exceed its number of antennas, the second constraint of (14) should be met:

(14) $$\begin{array}{c} \left\{\begin{array}{c} \sum_{i\in C(m)}al_{i,j}\left(t\right)\leq \sum_{i\in C(m)}il{}_{i,j}\left(t\right)\\ N_{a}\left(j\right)\geq \sum_{i=1:N}\left(\lambda {\;}_{i,j}\left(t\right)+il{}_{i,j}\left(t\right)-al_{i,j}\left(t\right)\right) \end{array}\right.,\left(j=St\right) \end{array}$$

Symbol $il_{i,j}\left(t\right)$ is the number of interference caused by vehicle *i* to station *j* and $al_{i,j}\left(t\right)$ is the number of aligned interference. Then we get

(15) $$al_{i,j}\left(t\right)\leq il_{i,j}\left(t\right),\left(IL\left(i,j\right)=1,j=St\right)$$

According to the meaning of $il_{i,j}\left(t\right)$, when vehicle is the off state that not transmits nor receives, it will not cause or receive any interference, we get

(16) $$il_{i,j}\left(t\right)=0,\left(\delta {\;}_{R}^{j}=0,j=St\right)$$

If vehicle is outside interference range of others, it will not be interfered by them.

(17) $$il{}_{i,j}\left(t\right)=0,\left(j=St,IL\left(i,j\right)=0\right)$$

For base station, signals are treated as interference if not intended for it. Thus, we have following constraint

(18) $$il{}_{i,j}\left(t\right)\leq \delta {\;}_{T}^{i}\left(t\right)\cdot \delta {\;}_{R}^{j},\left(j=St,\lambda {\;}_{i,j}\left(t\right)=0\right)$$

### 3.3. The Model of Data-Driven Assisted Transmission

In contrast to other types of information, safety related information in VANETs is open to all vehicles. Furthermore, during the same scheduling frame, information of a vehicle remains unchanged. Therefore, when a vehicle receives information from others, it could re-encode safety related information together and broadcasts to others in one session to improve the efficiency of information propagation. Based on these characteristics, in this subsection, we design a data-driven assisted transmission model for safety related information.

**Figure 6:**
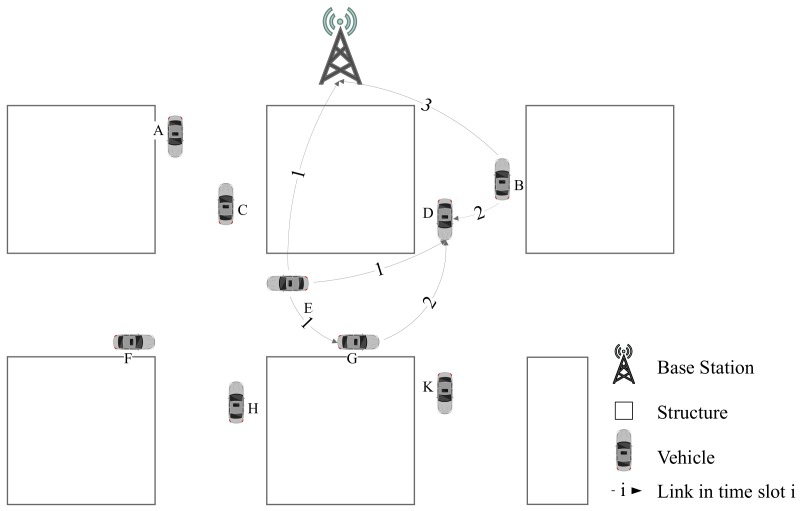
Example of V2I without Assisted Transmission.

Transmission without data-driven assisted is illustrated in Figure 6. Taking the session sent from vehicle E→D as an example, it can be delivered or relayed by base station and vehicle *G*. When vehicle *E* and *B* broadcast sessions simultaneously, vehicle *D* would receive two messages at same time slot. Once the number of received signals exceeds number of vehicle *D*’s freedom degrees, it will not be able to obtain the valid information. Therefore, in order to avoid collision, messages of vehicle *E* and vehicle *B* should be transmitted in different time slots. Since vehicle *G* also needs to transmit data to vehicle *D*, there should be one more time slot be assigned.

**Figure 7:**
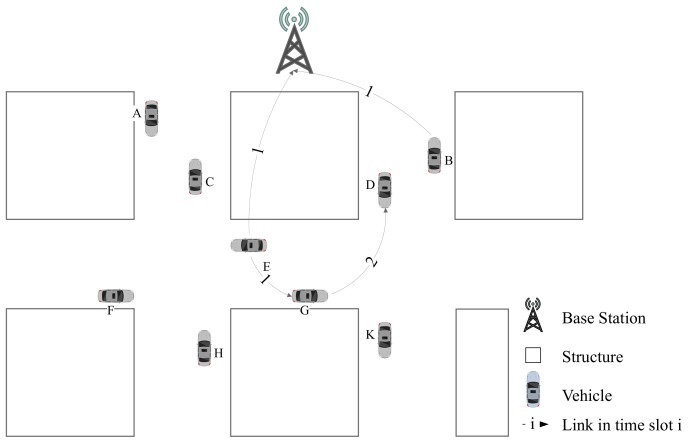
Example of V2I with Assisted Transmission.

As shown in Figure 7, we can arrange one time slot for session E→G with session B→I and another time slot for G→D. After vehicle *G* receives message from vehicle *E*, it can merge its own information with other vesicles. Although vehicle *D* get message of *E* one time slot later, there are only two time slots are occupied for all network.

Nevertheless, when vehicles adopt assisted transmission, it is necessary to ensure safety related information is delivered in time. In this paper, we choose minimum hop counts donated as hcount to be the upper limit of hops, then we get

(19) $$\sum_{i=1:N,j=1:N}rot{}_{i,j}\left(f\right)\leq hcount,\;\left(1\leq f\leq F\right)$$

$rot_{i,j}\left(t\right)$ is feasible routing path of session *f*. If a routing path bears a data relay task, the following formula is met

(20) $$rot{}_{i,j}\left(f\right)\leq \sum_{t=1:K}\lambda {\;}_{i,j}\left(t\right),\;\left(i=Src_{f},1\leq f\leq F\right)$$

For session *f*, its initiator is start of route, then we get

(21) $$\sum_{j=1:N}rot{}_{j,i}\left(f\right)\leq 0,\;\left(i=Src_{f},1\leq f\leq F\right)$$

(22) $$\sum_{j=1:N}rot{}_{i,j}\left(f\right)\geq 1,\;\left(i=Src_{f},1\leq f\leq F\right)$$

For relaying vehicles, we get the following constraint:

(23) $$\begin{array}{c} \sum_{j=1:N}rot{}_{i,j}\left(f\right)=\sum_{j=1:N}rot_{j,i}\left(f\right),\left(i\neq Src_{f},i\neq Des_{f}\right) \end{array}$$

As destination vehicles, there must be some vehicle that broadcasts message to them, then we have

(24) $$\sum_{j=1:N}rot{}_{j,i}\left(f\right)\geq 1,\;\left(i=Des_{f},1\leq f\leq F\right)$$

In order to avoid routing loop at destination vehicle, we have:

(25) $$\sum_{j=1:N}rot{}_{i,j}\left(f\right)\leq 0,\;\left(i=Des_{f},1\leq f\leq F\right)$$

### 3.4. Optimization Objective

It is obvious that the safety related information should be transmitted punctually. During each scheduling frame, we have *K* time slots and TU(t) is the state of time slot *t*. If there is no vehicle initiates communication at time slot *t*, TU(t) equals 0, then we get

(26) $$\left\{\begin{array}{c} TU\left(t\right)=1,\left(\sum_{i=1:N}\delta {\;}_{T}^{i}\left(t\right)\geq 1\right)\\ TU\left(t\right)=0,\left(\sum_{i=1:N}\delta {\;}_{T}^{i}\left(t\right)=1\right) \end{array}\right.,\;\left(1\leq t\leq K\right)$$

Fewer time slots are occupied, more efficient of safety related information propagation is. Therefore, we take time slot taken-up ratio denoted as PTU to be the optimization objective, then we get

(27) $$PTU=\sum_{t=1:K}TU(t)/K$$

Combined with other constraints, we have the optimization problem OPT–PTU:
**OPT:****Min**PTU
s.t.(1)–(3),(5),(7)–(27)

Among all the constraints, Formula (1)–(2) regulate half-duplex mode of vehicle terminals; (5)–(7) give constraints on receiving state and communication links; (8)–(10) introduce constraints on V2V broadcast communication; (11) presets initialization values to reduce searching range of feasible solutions; (14)–(18) regulate V2I and IA constraints for base station. (19)–(25) impose constraints on communicate route and data-driven assisted transmission. Among them, $\lambda_{i,j}\left(t\right),il_{i,j}\left(t\right),al_{i,j}\left(t\right)$ and $rot_{i,j}\left(t\right)$ are positive integer variables. PTU is non-negative variable. hcount can be adjusted according to delay requirement of VANETs.

Due to type of variable TU(t) and constraint (18) which is not linear, the prime optimization problem is Mixed Integer Nonlinear Programming (MINLP). In order to solve this problem, we have to reform some constraints into much neater forms. The next two lemmas will help us to reduce complexity of OPT–PTU problem. Proofs of these two lemmas are postponed to Appendix A.

**Lemma** **1.** *Constraint (26) with integer variable TU(t) is identical with the form listed in (28)*

(28) $$\left\{\begin{array}{c} TU\left(t\right)\leq 1-1/N+\sum_{i=1:N}\delta {\;}_{T}^{i}\left(t\right)/N\\ TU\left(t\right)\geq \sum_{i=1:N}\delta {\;}_{T}^{i}\left(t\right)/N \end{array}\right.$$

**Lemma** **2.** *Nonlinear constraint (16) combined with (17), (18) can be reformed in to the following linear constraint (29)–(31)*

(29) $$il{}_{i,j}\left(t\right)\geq \delta {\;}_{T}^{j}\left(t\right)+\delta {\;}_{R}^{i}\left(t\right)-\lambda {\;}_{i,j}\left(t\right))-1,\left(IL\left(i,j\right)\neq 0\right)$$

(30) $$il{}_{i,j}\left(t\right)\leq \delta {\;}_{R}^{i}\left(t\right),\left(IL\left(i,j\right)\neq 0\right)$$

(31) $$\left\{\begin{array}{c} il{}_{i,j}\left(t\right)\leq 1-\lambda {\;}_{i,j}\left(t\right)\\ 0\leq il{}_{i,j}\left(t\right) \end{array}\right.,\left(IL\left(i,j\right)\neq 0\right)$$

With these two lemmas, prime optimization problem is identical with the following
**OPT:****Min**PTU
s.t.(1)–(3),(5),(7)–(15),(19)–(25),(27)–(31)

OPT–PTU is reformed into Mixed Integer Linear Programming that can be solved more efficiently compared with prime one.

## 4. The σ-Clustering Algorithm Based on CSI in VANET

To solve CSI stability problem, which is the key information of IA application, in this section, we design a CSI based clustering algorithm. Reasonable CSI clustering algorithm would improve accuracy of CSI estimation and IA efficiency though avoiding vehicles with similar CSI allocated the same time slot. In this paper, we take RSSI value as CSI parameter.

$C=\{C_{1},C_{2},\ldots ,C_{\eta }\}$ is the clustered vehicle set. η=∣C∣ is the number of clusters. $p_{i}$ is RSSI value between vehicle *i* and base station. $\varepsilon_{i}$ is variance of each cluster which is calculated as follows

(32) $$\varepsilon_{j}=\sum_{i\in C_{j}}\left|\right|p_{i}-\frac{\sum_{m\in C_{j}}p_{m}}{∥C_{j}∥}|{|}^{2},\left(1\leq j\leq \eta \right)$$

Traditional clustering algorithms is set with fixed η. In our clustering algorithm, we impose requirement on $\varepsilon_{i}$. Define upper limit of $\varepsilon_{i}$ as σ. Then, we have

(33) $$\varepsilon_{i}\leq \sigma \;,\;\left(\varepsilon_{i}\in \varepsilon \right)$$

According to the definition of $\varepsilon_{i}$, we can conclude that the number of clusters increases while the value of σ decreases. A reasonable value of σ should be determined.

Since signal fading rate varies due to buildings and other obstacles, the clustering algorithm only based on RSSI is not pragmatic in VANETs. Hence, the location information should also be taken into consideration. After RSSI based clustering algorithm, we introduce kemans++ clustering algorithm based on location to avoid this problem. Pseudocode of our algorithm is listed in Algorithm 1.

**Algorithm 1** Clustering (A,St,ε,σ)
1:set the max cluster number η2:Clusters, Clusters23:
N=size(A)
4:
PathLosses=CalPathLoss(A,St)
5:**for**i=1 to η **do**6: $\left\{C_{1},C_{2}\ldots C_{i}\right\}$ = Cluster Based on Path loss7: $\left\{\varepsilon_{1},\varepsilon_{2},\ldots ,\varepsilon_{i}\right\}$ = Calculate ε for each cluster8: **for** j=1 to *i* **do**9:  **if** $\varepsilon_{j}\leq \sigma$ **then**10:   Add the $C_{j}$ into Clusters11:  **end if**12:  **if** $\varepsilon_{j}\geq \sigma$ **then**13:   continue14:  **end if**15: **end for**16:
**end for**
17:**for***c* in $\left\{C_{1},C_{2}\ldots C_{i}\right\}$ **do**18: cluster2=Kmeans++(*c*) based on location of vehicles19: Add cluster2 into Clusters220:
**end for**
21:
**return** Clusters2



## 5. Simulations

### 5.1. Simulation for Clustring Algorithm

**Figure 8:**
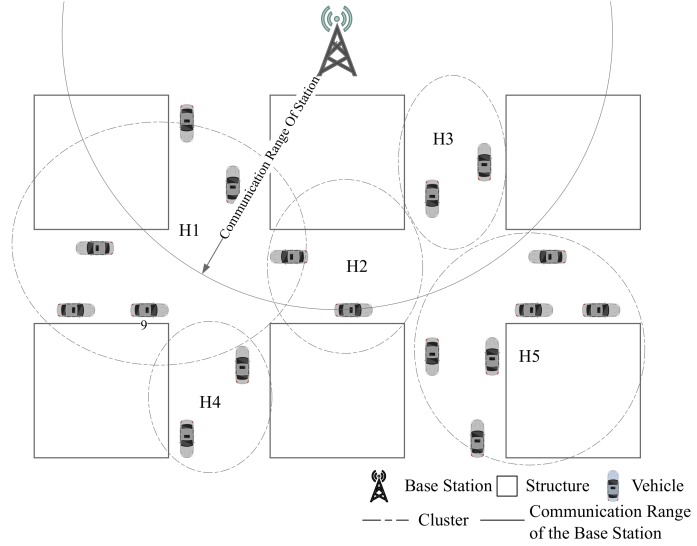
Base Station and Clusters ($H_{i}$ indicates cluster of vehicles with similar CSI).

Our simulation scenario is shown in Figure 8, including 7 vertical streets and 3 horizontal streets in this scenario. Vehicles are generated by SUMO [13]. Simulation area is 500 m in width and 2000 m in length. There is one base station in this area. The time slots of each scheduling frame is set to 20. Gurobi is chosen to be the solver for the optimization [26].

**Figure 9:**
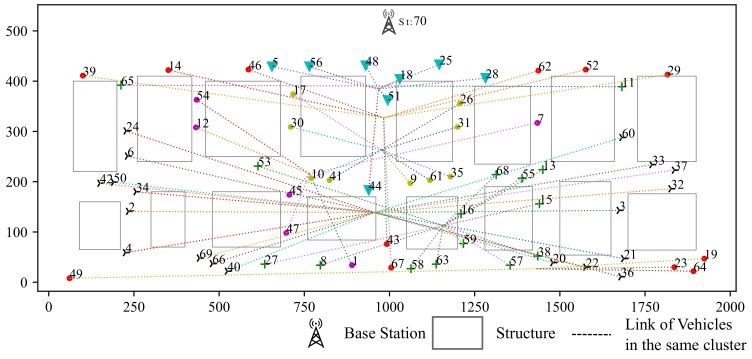
Clustering According to RSSI.

Clustering results based on RSSI and location are shown in Figure 9. The RSSI based clustering vehicles are grouped into ring-shaped area which is obviously not suitable for our scenarios since it does not take buildings into consideration.

**Figure 10:**
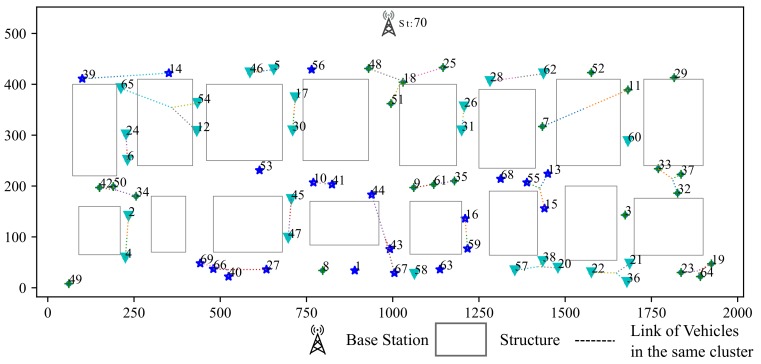
σ-Clustering Algorithm According to path loss and location.

The clustering result based on σ-clustering algorithm is shown in Figure 10. Vehicles from the same intersection or blocked by the same building are grouped into the same cluster, which is more reasonable.

### 5.2. Analysis of IA Simulation

In our simulation, hcount is set to 6 to limit the number of hops in data routing. Then we analyze simulation results based on V2V, I2V and V2I cases.

**Figure 11:**
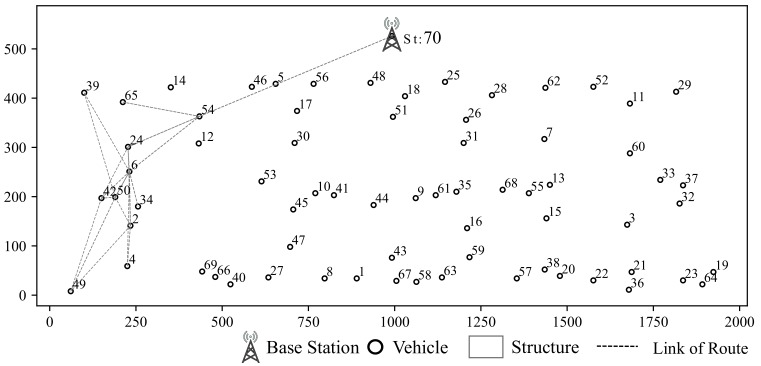
Example of V⇌V Communication Simulation Result.

**Case Study for V2V communication:** As shown in Figure 11, we select some vehicles as example. The communication sessions of selected vehicles are listed in Table 2 and routes of some sessions are listed in Table 3. From Table 3, we find that only 5 sessions adopt direct communication. And vehicle 6 and 54 contribute most routing tasks. It is because that vehicle 54 is closer to base station than any other vehicles in the same cluster and vehicle 6 is located in the center of its cluster.

**Figure 12:**
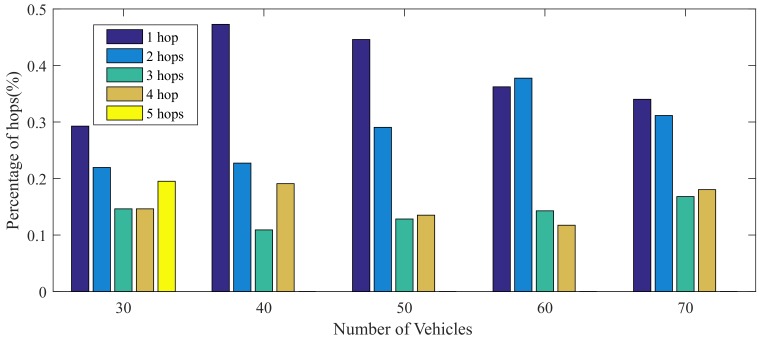
Graph of Proportions of Different Hops.

The hop counts are shown in Table 4 with different size of experiments. The 5-hop routing only occurs when there are 30 vehicles in this road since the distribution of these cars is sparser. The percentage of different hop counts are shown in Figure 12. With the vehicle density increasing, the percentage of two hops is increased and the direct communication is reduced, which means the information would be propagated through assisted transmission instead of directly communication under the condition with more vehicles.

**Figure 13:**
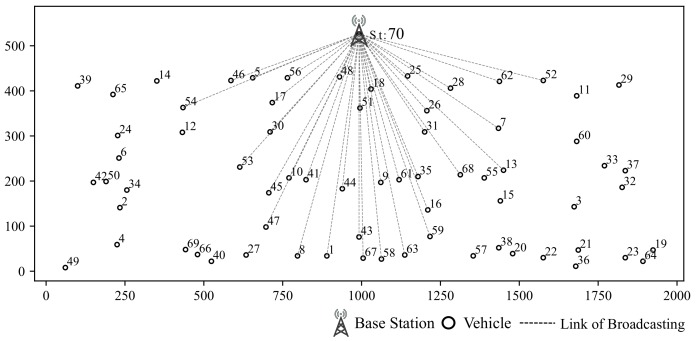
Sessions Base Station Participated.

**Figure 14:**
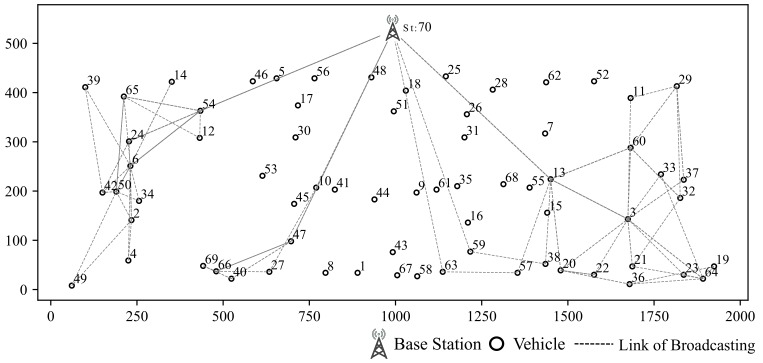
Sessions Uploading from Vehicles out of Base Station Communication Range.

**Case Study for I2V Assisted Communication:** If a vehicle is within communication range of base station, direct data transmission is preferred. Otherwise, multi-hop forwarding will be adopted mostly. Direct communication from base station is shown in Figure 13 and vehicles out of communication range choose multi-hop communication method as shown in Figure 14. As increase in number of vehicles, the base station takes part in assisting data forwarding more frequently due to its higher concurrent communication ability as shown in Table 5.

**Figure 15:**
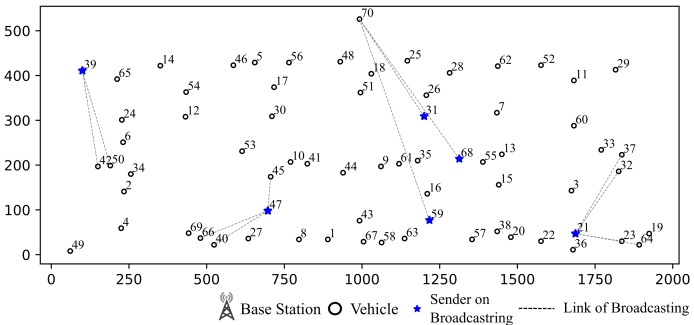
Example of IA in 20th Time Slot.

**Case Study for IA in V2I Communication:** During the 20th time slot, vehicle 21, 31, 39, 47, 59, 68 broadcast message as shown in Figure 15. Vehicle 31, 68 and 59 broadcast to base station that will take up 3 antennas of the base station. However, the base station is also in the interference range of vehicle 39 and 47. Therefore, in order to receive intended messages from 31, 68 and 59, the interference caused by 39 and 47 will be aligned together to save 1 degree of communication freedom.

**Figure 16:**
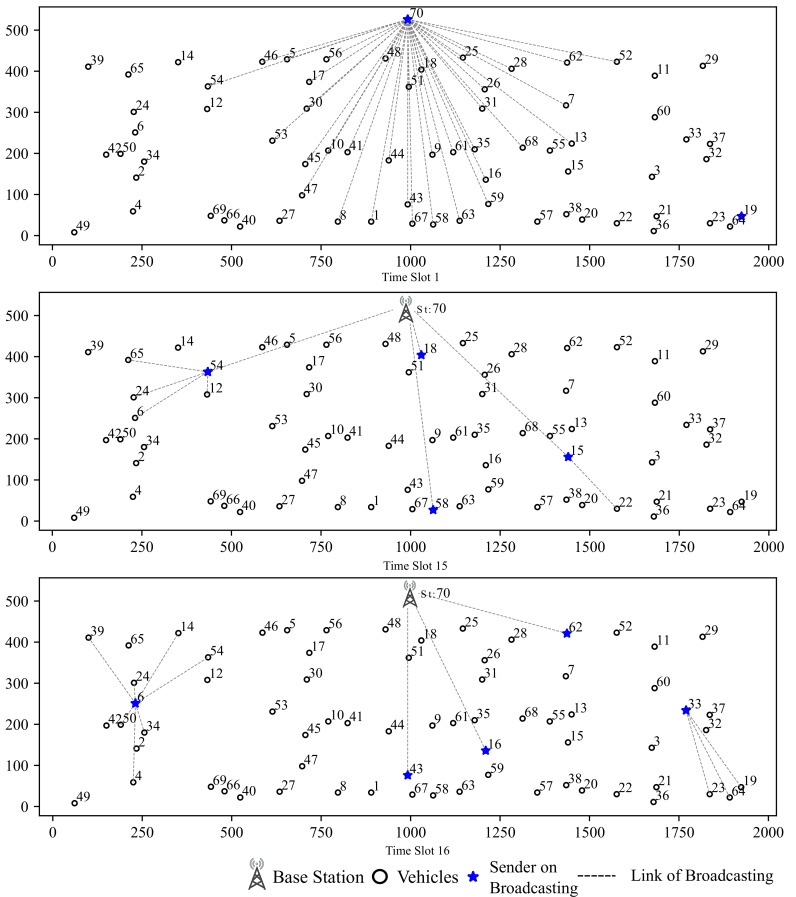
An Example of I→V Session Routes from Base Station to Vehicles out of Vommunication Range.

**Case Study for Data-driven Assisted Transmission:** We draw an example of data routing path as shown in Figure 16. The route table is shown in Table 6:

As shown in Figure 16, during 1st slot, the base station initiates a broadcast and vehicle 54 receives the message from base station. At time slot 15, vehicle 54 broadcasts to its surrounding vehicles. Since vehicle 65, 24 and 6 are out of the base station’s communication range, the session from base station is relayed by vehicle 54. At time slot 16, vehicle 6 center on the cluster broadcasts messages to surrounding vehicles. In this time slot, vehicle 6 relays message sent from vehicle 54 at time slot 15. To make full use of channel resources, during time slot 16, vehicles 43, 16 and 62 are assigned to send messages to base station simultaneously and vehicle 33 broadcasts to surrounding vehicles.

**Figure 17:**
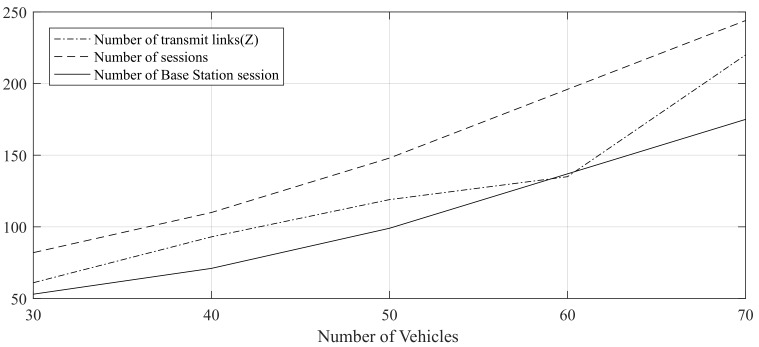
Number of Route Links and Sessions.

The experiment results listed in Table 7 and Figure 17 show that there exists approximate linearity between number of sessions and routing links with number of vehicles.

**Figure 18:**
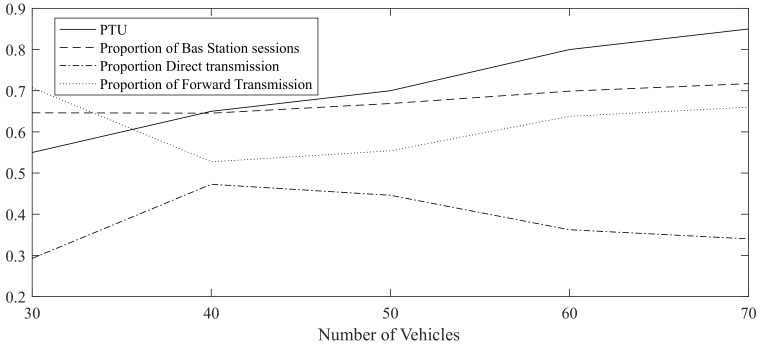
PTU and Some Percentages of Sessions as the number of vehicles increases.

As shown in Figure 18, with the increase in the number of vehicles, proportion of routing task that base station participated also increases, which is is due to MIMO and large broadcast coverage of base station. Besides, PTU keeps increasing along with increase of vehicle number. The proportion of idle time slots as shown in Table 8, could be 45% at 30 vehicles. Even in 70 vehicles simulation, this number could keep 15%. For comparisons, the time slots of MIMO without IA are almost used up when there are 70 vehicles, which would lead communication congestion.

As shown in Table 9, in simulation with 30 vehicles, the proportion direct transmission is relatively high, mainly due to fewer vehicles within safety distance. And communication delay could be reduced by 45%. However, with the increase of vehicle number, the proportion of assisted transmission is getting higher, which can reach 50%. Routing communication is reduced by an average of about 20% compared with no assisted transmission.

## 6. Conclusions

This paper studies communication problems of safety related information in VANETs. Under the proposed framework, the IA technology applied in VANETs is realized. Taking advantage of data-driven assisted transmission, the use ratio of route is improved. The simulation results show that this approach can effectively improve VANETs link use and reduce communication delay.

## Figures and Tables

**Table 1:** SYMBOLS and NOTATION.

Symbol	Remark
*N*	Number of terminals in network including vehicles and base station
*F*	Number of communication times
St	ID of Base station
$Src=\left\{Src{}_{1},Src_{2}...Src_{F}\right\}$	Set of sources
$Des=\left\{Des{}_{1},Des_{2}...Des_{F}\right\}$	Set of destinations
$Ts=\left\{t_{1},t_{2}...t_{K}\right\}$	Set of Scheduling time slots
*K*	Number of time slots
$N_{a}\left(i\right)$	Number of antennas equipped on terminal *i*
IL(i,j)	Symbol of interference status between *i* and *j* (0 means *j* is out of interference range of *i*)
L(i,j)	Symbol of connectivity between *i* and *j* (0 means *j* is out of communicate range of *i*)
C(i)	Set of vehicles in cluster *i*
$\lambda {\;}_{i,j}\left(t\right)$	Number of communication session vehicle *i* sends to vehicle *j* during the same time slot *t*
$il_{i,j}\left(t\right)$	Number of interference caused by *i* to *j* during time slot *t*
$al_{i,j}\left(t\right)$	Number of interference caused by *i* to *j* which could be aligned during time slot *t*
$\delta {\;}_{T}^{i}\left(t\right)$	Sending state of vehicle *i* at time slot *t* (0 means that vehicle *i* is not broadcasting during time slot *t*)
$\delta {\;}_{R}^{i}\left(t\right)$	Receiving state of vehicle *i* at time slot *t* (0 means that vehicle *i* is not receiving during time slot *t*)
hcount	Threshold value of max hops
$rot_{i,j}\left(f\right)$	Integer value indicates whether vehicle *i* relays data to *j* for session *f* (1 means that *i* and *j* take part in relaying for session *f* )
TU(t)	Integer value indicates whether time slot *t* is assigned to any vehicles (0 means that there is no vehicle sends data during time slot *t*, 1 means that at least one vehicle sends data to others)
PTU	Proportion of the time slots allocated

**Table 2:** Src and Des for V⇌V Session.

SRC	DES 1	DES 2	DES 3	DES 4	DES 5
65	24	70			
24	6	65	70		
6	24	34	42	50	70
50	2	6	34	42	70
42	6	50	70		
37	32	33	70		
2	4	34	50	70	
4	2	70			

**Table 3:** Routes of V⇌V.

SRC	DES	ROUTE	SRC	DES	ROUTE
65	24	54	50	34	6
24	6	2	50	42	
24	65	54	42	6	
6	24		42	50	49
6	34		2	4	6
6	42	50	2	34	6
6	50	39	2	50	49
50	2	4			
50	6				

**Table 4:** Number of Route Hops for V⇌V as The Number of Vehicles Increases.

	Number of Hops	1	2	3	4	5
Number of Vehicle						
30		24	18	12	12	16
40		52	25	12	21	0
50		66	43	19	20	0
60		71	74	28	23	0
70		83	76	41	44	0

**Table 5:** Percentage of Base Station Participation in Route.

Number of Vehicles	Number of all Sessions	Count of Participation of Base Station	Percentage
30	82	5	0.06
40	110	14	0.13
50	148	21	0.14
60	196	45	0.23
70	244	50	0.20

**Table 6:** Proportions of Different Hops in Routes of V⇌V.

Src	Des	Routes	Src	Des	Routes
70	65	54	70	14	54, 6
70	24	54	70	50	54, 6
70	6	54	70	4	54, 6
70	39	54, 6	70	34	54, 6

**Table 7:** The number of Sessions and Transmit Links.

Number of Vehicles	Number of Transmit Links (Z)	Number of Sessions	Number of Base Station Session
30	61	82	53
40	93	110	71
50	119	148	99
60	135	196	137
70	220	244	175

**Table 8:** PTU and Percentages of Sessions.

Number of Vehicles	Proportion of Base Station Session	Ratio of Transmit Links to Sessions	PTU	PTU without IA
30	0.65	0.74	0.55	0.65
40	0.65	0.85	0.65	0.76
50	0.67	0.80	0.70	0.88
60	0.70	0.69	0.80	0.93
70	0.72	0.90	0.85	0.98

**Table 9:** Proportion of Direct and Forward Transmission.

Number of Vehicles	Proportion Direct Transmission	Proportion of Forward Transmission
30	0.30	0.71
40	0.47	0.53
50	0.45	0.55
60	0.36	0.64
70	0.34	0.66

## Abbreviations

The following abbreviations are used in this manuscript:

VANETs	Vehicular Ad-hoc NETworks
IA	Interference Alignment
V2V	Vehicle to Vehicle and Vehicle
V2I	Vehicle to Infrastructure
CSI	Channel State Information
CR	Cognitive Radio
SD	Spectrum Database
TDMA	Time Division Multiple Access
ITS	Intelligent Transportation Systems
MIMO	Multiple-Input Multiple-Output
MINLP	Mixed Integer Nonlinear Programming

## Appendix A

**Proof** **of** **Lemma** **1.** Constraint (26) with integer variable TU(t) is identical with the form listed in (28). By the definition of TU(t), if there exists any data transmission in time slot t, then TU(t) equals to 1, otherwise, it is 0. (1) If there exists any data transmission in time slot *t*, then there must be one $\delta {\;}_{T}^{i}\left(t\right)$ that not equals 0. Therefore, we have

$$\sum_{i=1:N}\delta {\;}_{T}^{i}\left(t\right)\geq 1$$

For the first inequality in (28), we have:

$$TU\left(t\right)\leq 1-1/N+\sum_{i=1:N}\delta {\;}_{T}^{i}\left(t\right)/N<2$$

where $\sum_{i=1:N}\delta {\;}_{T}^{i}\left(t\right)/N$ is greater than or equal to 1. Therefore, we have TU(t) is less than or equal to α which is greater than or equal to 1. For the second inequality in (28), we have:

$$TU\left(t\right)\geq \sum_{i=1:N}\delta {\;}_{T}^{i}\left(t\right)/N=1/N>0$$

Combining the above, we have:

1/N≤TU(t)<2

Since the value of TU(t) can only be chosen from integer 0 and 1, we have TU(t)=1, which is identical to the first equation in (28) (2) If there is no data transmission on time slot t, it is obvious that $\sum_{i=1:N}\delta {\;}_{T}^{i}\left(t\right)$ equals 0. For the first inequality in (28), we have:

$$TU\left(t\right)\leq 1-1/N+\sum_{i=1:N}\delta {\;}_{T}^{i}\left(t\right)/N=1-1/N<1$$

For the second inequality in (28), we have:

$$TU\left(t\right)\geq \sum_{i=1:N}\delta {\;}_{T}^{i}\left(t\right)/N=0$$

Combining the above, we have:

0≤TU(t)≤1−1/N<1

Since the value of TU(t) can only be chosen from integer 0 and 1, we have TU(t)=0, which is identical to the second equation in (28). Therefore, we draw the conclusion that (26) and (28) are identical to each other. □

**Proof** **of** **Lemma** **2.** Nonlinear constraint (16) combined with (17), (18) can be reformed in to the following linear constraint (29)–(31) During the communication, we have following cases:

(1) When vehicle *i* is in transmitting mode, we have $\delta_{T}^{i}\left(t\right)=1$. In the same time slot, if vehicle *j* is in receiving mode, we have $\delta_{R}^{j}\left(t\right)=1$. However, if session from vehicle *i* is NOT intended for *j*, we have $\lambda_{i,j}\left(t\right)=0$. In this case, we have $il_{i,j}\left(t\right)=0$.
(2) When vehicle *i* is in transmitting mode, we have $\delta_{T}^{i}\left(t\right)=1$. In the same time slot, if vehicle *j* is in receiving mode, we have $\delta_{R}^{j}\left(t\right)=1$. And if session from vehicle *i* is intended for *j*, we have $\lambda_{i,j}\left(t\right)=1$. In this case, we have $il_{i,j}\left(t\right)=0$.
(3) When vehicle *i* is in transmitting mode, we have $\delta_{T}^{i}\left(t\right)=1$. In the same time slot, if vehicle *j* is NOT in receiving mode, we have $\delta_{R}^{j}\left(t\right)=0$. In this case, we have $\lambda_{i,j}\left(t\right)=0$. In this case, we have $il_{i,j}\left(t\right)=0$.
(4) When vehicle *i* is NOT in transmitting mode, we have $\delta_{T}^{i}\left(t\right)=0$. In the same time slot, if vehicle *j* is in receiving mode, we have $\delta_{R}^{j}\left(t\right)=1$. In this case, we have $\lambda_{i,j}\left(t\right)=0$. In this case, we have $il_{i,j}\left(t\right)=0$.
(5) When vehicle *i* is NOT in transmitting mode, we have $\delta_{T}^{i}\left(t\right)=0$. In the same time slot, if vehicle *j* is in receiving mode, we have $\delta_{R}^{j}\left(t\right)=0$. In this case, we have $\lambda_{i,j}\left(t\right)=0$. In this case, we have $il_{i,j}\left(t\right)=0$.

Combining the above five cases, we have the following Equation:

$$il{}_{i,j}\left(t\right)=\delta {\;}_{T}^{i}\left(t\right)\cdot \delta {\;}_{R}^{j}\cdot \left(1-\lambda {\;}_{i,j}\left(t\right)\right)=\delta {\;}_{T}^{i}\left(t\right)\cdot \delta {\;}_{R}^{j}-\delta {\;}_{T}^{i}\left(t\right)\cdot \delta {\;}_{R}^{j}\cdot \lambda {\;}_{i,j}\left(t\right)$$

We have two items on the right-hand-side of the equation. One is in the quadratic form, the other one is in cubic form, which will lead to a relatively high complexity in solving the optimization problem. What is done next is to reshape the polynomial form of constraint into a linear one. Considering the following constraints with (10), (29)–(31):

$$\left\{\begin{array}{c} il{}_{i,j}\left(t\right)\geq \delta {\;}_{T}^{j}\left(t\right)+\delta {\;}_{R}^{i}\left(t\right)-\lambda {\;}_{i,j}\left(t\right))-1,\;(IL\left(i,j\right)\neq 0\\ il{}_{i,j}\left(t\right)\leq \delta {\;}_{T}^{i}\left(t\right)\cdot N_{a}\left(i\right),\;\left(j\notin St\right)\\ il{}_{i,j}\left(t\right)\leq \delta {\;}_{R}^{i}\left(t\right),\;(IL\left(i,j\right)\neq 0\\ \left\{\begin{array}{c} il{}_{i,j}\left(t\right)\leq 1-\lambda {\;}_{i,j}\left(t\right)\\ 0\leq il{}_{i,j}\left(t\right) \end{array}\right.,\;\left(IL\left(i,j\right)\neq 0\right) \end{array}\right.$$

In the case corresponding to case (1), we have:

$$\delta {\;}_{T}^{i}\left(t\right)=1,\delta {\;}_{R}^{j}\left(t\right)=1,\lambda {\;}_{i,j}\left(t\right)=1$$

$$\left\{\begin{array}{c} il_{i,j}\left(t\right)\geq 1+1-1-1=0\\ il_{i,j}\left(t\right)\leq 1\\ il_{i,j}\left(t\right)\leq 1\\ il_{i,j}\left(t\right)\leq 1-1=0 \end{array}\right.\Rightarrow \lambda {\;}_{i,j}\left(t\right)=0$$

In the case corresponding to case (2), we have:

$$\delta {\;}_{T}^{i}\left(t\right)=1,\delta {\;}_{R}^{j}\left(t\right)=1,\lambda {\;}_{i,j}\left(t\right)=0$$

$$\left\{\begin{array}{c} il_{i,j}\left(t\right)\geq 1+1-0-1=1\\ il_{i,j}\left(t\right)\leq 1\\ il_{i,j}\left(t\right)\leq 1\\ il_{i,j}\left(t\right)\leq 1-0=1 \end{array}\right.\Rightarrow \lambda {\;}_{i,j}\left(t\right)=1$$

In the case corresponding to case (3), we have:

$$\delta {\;}_{T}^{i}\left(t\right)=0,\delta {\;}_{R}^{j}\left(t\right)=0,\lambda {\;}_{i,j}\left(t\right)=0$$

$$\left\{\begin{array}{c} il_{i,j}\left(t\right)\geq 0+0-0-1=-1\\ il_{i,j}\left(t\right)\leq 0\\ il_{i,j}\left(t\right)\leq 0\\ il_{i,j}\left(t\right)\leq 1-0=1 \end{array}\right.\Rightarrow \lambda {\;}_{i,j}\left(t\right)=0$$

In the case corresponding to case (4), we have:

$$\delta {\;}_{T}^{i}\left(t\right)=0,\delta {\;}_{R}^{j}\left(t\right)=1,\lambda {\;}_{i,j}\left(t\right)=0$$

$$\left\{\begin{array}{c} il_{i,j}\left(t\right)\geq 0+1-0-1=0\\ il_{i,j}\left(t\right)\leq 0\\ il_{i,j}\left(t\right)\leq 1\\ il_{i,j}\left(t\right)\leq 1-0=1 \end{array}\right.\Rightarrow \lambda {\;}_{i,j}\left(t\right)=0$$

In the case corresponding to case (5), we have:

$$\delta {\;}_{T}^{i}\left(t\right)=1,\delta {\;}_{R}^{j}\left(t\right)=0,\lambda {\;}_{i,j}\left(t\right)=0$$

$$\left\{\begin{array}{c} il_{i,j}\left(t\right)\geq 1+0-0-1=0\\ il_{i,j}\left(t\right)\leq 1\\ il_{i,j}\left(t\right)\leq 0\\ il_{i,j}\left(t\right)\leq 1-0=1 \end{array}\right.\Rightarrow \lambda {\;}_{i,j}\left(t\right)=0$$

Therefore, the outcomes of these series of inequalities are identical to the values calculated by Equation (10), (29)–(31). Hence, we reach the conclusion that polynomial constraint (16), (17), (18) is the same as inequalities (10), (29)–(31). □
